# Phylogenetic Clustering of Origination and Extinction across the Late Ordovician Mass Extinction

**DOI:** 10.1371/journal.pone.0144354

**Published:** 2015-12-14

**Authors:** Andrew Z. Krug, Mark E. Patzkowsky

**Affiliations:** Department of Geosciences, The Pennsylvania State University, University Park, Pennsylvania, United States of America; University of Oxford, UNITED KINGDOM

## Abstract

Mass extinctions can have dramatic effects on the trajectory of life, but in some cases the effects can be relatively small even when extinction rates are high. For example, the Late Ordovician mass extinction is the second most severe in terms of the proportion of genera eliminated, yet is noted for the lack of ecological consequences and shifts in clade dominance. By comparison, the end-Cretaceous mass extinction was less severe but eliminated several major clades while some rare surviving clades diversified in the Paleogene. This disconnect may be better understood by incorporating the phylogenetic relatedness of taxa into studies of mass extinctions, as the factors driving extinction and recovery are thought to be phylogenetically conserved and should therefore promote both origination and extinction of closely related taxa. Here, we test whether there was phylogenetic selectivity in extinction and origination using brachiopod genera from the Middle Ordovician through the Devonian. Using an index of taxonomic clustering (R_CL_) as a proxy for phylogenetic clustering, we find that A) both extinctions and originations shift from taxonomically random or weakly clustered within families in the Ordovician to strongly clustered in the Silurian and Devonian, beginning with the recovery following the Late Ordovician mass extinction, and B) the Late Ordovician mass extinction was itself only weakly clustered. Both results stand in stark contrast to Cretaceous-Cenozoic bivalves, which showed significant levels of taxonomic clustering of extinctions in the Cretaceous, including strong clustering in the mass extinction, but taxonomically random extinctions in the Cenozoic. The contrasting patterns between the Late Ordovician and end-Cretaceous events suggest a complex relationship between the phylogenetic selectivity of mass extinctions and the long-term phylogenetic signal in origination and extinction patterns.

## Introduction

Mass extinctions can change the trajectory of life by eliminating established lineages and allowing other taxonomic groups to diversify, but in some cases the effects of the extinction can be relatively small even when extinction rates are high [1–6]. For example, the Late Ordovician mass extinction is the second most severe in terms of the proportion of genera lost, whereas the end-Cretaceous ranks fifth. However, the Late Ordovician generally ranks last among the big five mass extinctions, and even beneath the far less severe Serpukhovian extinction, in terms of ecological severity (i.e. an estimation of the disruption or reorganization of ecosystems and clades) [6,7]. The end-Cretaceous, in contrast, ranks near the top of ecological severity, second only to the Late Permian mass extinction. This disconnect may arise because the intensity of a mass extinction is traditionally determined by counting taxa, typically the number of genera or families to go extinct [8], a metric that ignores the amount of evolutionary history removed by mass extinction. Evolutionary history refers to the length of the branches separating two taxa in a phylogenetic tree [9], and quantifies the amount of evolution that has occurred since the divergence of these taxa from their common ancestor. Random extinctions in a phylogenetic tree eliminate relatively little evolutionary history, even when extinction intensity is high, whereas the same level of extinction can remove much greater portions of evolutionary history when clustered (selective) [10,11]. Because phylogenetically selective extinctions can remove large amounts of evolutionary history at once, the degree to which extinctions are phylogenetically selective could explain the disconnect between the severity of an extinction event and its effect on the history of life [6,7]. Accounting for evolutionary history in studies of mass extinction and recovery [12] can also add insight into the factors driving diversification, many of which may be phylogenetically conserved (i.e. likely to be passed from parent to daughter species) [12–16]. A more complete understanding of these factors requires comparative studies between different events, time intervals, and taxa.

Here, we test for phylogenetic selectivity in brachiopod extinction and origination for time intervals ranging from the Middle Ordovician through the Devonian, an interval that encompasses the Late Ordovician mass extinction, and compare these results to prior work on phylogenetic patterns of extinction for Mesozoic and Cenozoic bivalves [12], including the End Cretaceous mass extinction. We find that family membership predicts extinction and origination in the Silurian and Devonian, but the signal is much weaker in the Ordovician, including the Late Ordovician mass extinction, suggesting that a major shift in the nature of diversification was established in the recovery interval and lasted for tens of millions of years. Our results stand in stark contrast to phylogenetic extinction patterns for bivalves before and after the end-Cretaceous mass extinction [12], supporting the link between the level of phylogenetic clustering of mass extinctions and their ecological severity.

## Materials and Methods

### Brachiopod Taxonomic and Stratigraphic Data

We combined a database on the first and last appearance of all brachiopod genera [17] with an up-to-date taxonomic framework to determine the phylogenetic structure of extinctions and originations for stratigraphic intervals spanning the Late Ordovician through the Devonian. First and last appearances were binned into time intervals with an average duration of ~10 Myr in order to maintain adequate samples sizes for analysis. Stratigraphic ranges were ranged through to the beginning or end of bins in which their first or last appearance occurred. We also performed analyses by binning data into ~5 myr intervals, which did not change the results.

We used genera for our analysis because brachiopod genera have a relatively stable taxonomic framework and their stratigraphic ranges are better known than their constituent species. The family and superfamily assignments for genera were made using the Treatise on Invertebrate Paleontology [18–20], which, in the absence of a genus-level phylogeny of the phylum, serves as a recent, standardized treatment of brachiopod taxonomy and evolutionary relationships. All subgenera were raised to the status of genus for the analysis. Families were chosen to investigate phylogenetic clustering because they offer the finest taxonomic resolution possible while maintaining adequate sample sizes within intervals.

Because little modern phylogenetic work has been done on brachiopods, we use taxonomic membership as a proxy for phylogenetic relationships. Although not ideal, several studies have shown that taxonomic frameworks can be used when phylogenies based on modern methods are not available. For example, Congreve et al [21] showed that many families of strophomenid brachiopods were monophyletic, though Carlson and Fitzgerald [22] showed a lack of congruence between phylogenetic relationships and taxonomic groupings of genera and families for Devonian terebratulide brachiopods. A study on the relationship between extinction risk and various traits in the same group of Devonian terebratulide brachiopods [23] stressed the importance of incorporating phylogeny along with taxonomic relationships into the study, though they recovered consistent results using taxonomic and phylogenetic methods in the study, suggesting both methods produce similar results. Taxonomic groupings have also been used to estimate phylogenetic patterns of extinction in fossil bivalves [12] and in modern taxa, particularly among conservation biologists [24]. Jablonski and Finarelli [25] showed a general congruence between taxonomic groupings and phylogenetic relationships across a wide range of taxa, and Soul and Friedman [26] found that, under some circumstances, taxonomies, or trees that were produced from taxonomic information, could be used in place of cladistically derived phylogenies. Nonetheless, because brachiopod families may not all represent monophyletic groups [22, 27–28], we repeated the analysis at the coarser superfamily level. Results using both taxonomic levels were consistent (see *Results* section below), suggesting that error introduced through improper taxonomic assignments of genera to families does not bias the results. Though we believe the taxonomic metric used herein to be an adequate proxy for phylogenetic signal, the absence of a true phylogeny means our results more accurately describe clumping within taxonomic units rather than on a phylogenetic tree.

### Taxonomic Clustering

To compare our results on the Late Ordovician to the end-Cretaceous mass extinction, we followed the approach used by Roy et al. [12] in their study of end Cretaceous marine bivalves, though we applied their method to both extinction and originations as opposed to only extinctions. An index of taxonomic clustering (R_CL_; [12]) was calculated as the correlation between the elements of two matrices—(i) pairwise taxonomic similarities between all genera in an interval, with a value of 1 for genera in the same family and 0 for those in different families, and (ii) pairwise extinction/origination similarities of genera, with a value of 1 when two genera originate or go extinct in the same time interval and 0 otherwise. If extinctions or originations were taxonomically random, than R_CL_ should be near zero [12]. Positive values of R_CL_ indicate taxonomically clustered extinction/origination (i.e. extinction/origination are concentrated within some families more often then expected by chance), while negative values indicate extinction/origination dispersed among families. The significance of both positive and negative excursions of R_CL_ was determined by randomizing the genera within each interval that originated or went extinct, recalculating R_CL_, and repeating 1000 times. Only time intervals with more than 100 genera were used for the analyses, as small sample sizes can create artificially large shifts in both the R_CL_ values and the null expectation. Our data suggest no strong correlation (Table 1) between the R_Cl_ metric and raw diversity or turnover rates, including: diversity within a bin, number of extinctions/originations, proportional extinction/origination, or per-taxon extinction/origination [29].

**Table 1:** Lack of correlation between RCL and various diversity and turnover metrics. All correlations done using Spearman rank-order correlations. Results consistent regardless of test statistic.

	R_CL_ for Extinctions		R_CL_ for Originations	
	rho	p	Rho	p
Number of genera	-.203	.42	.249	.32
Number of extinctions	.007	.98	-	-
Number of originations	-	-	.356	.15
Proportion of genera extinct	.377	.12	-	-
Proportion of genera originating	-	-	.185	.46
Per-taxon extinction (q)	.53	.16	-	-
Per-taxon origination (p)	-	-	-.224	.37

We tested for clustering of extinction and origination in two ways. First, we performed an exact binomial test on Rcl for the complete data set to test whether there was a tendency for clustering (Rcl > 0) for all eighteen time intervals spanning 120 million years. Second, we tested for changes in Rcl through time by comparing Rcl in the Ordovician with Rcl in the Silurian-Devonian in order to see how the Late Ordovician affected clustering of origination and extinction. We performed a Komolgorov-Smirnov test comparing Rcl between intervals. We also performed an exact binomial test for each interval to determine if the number of Rcl values lying outside of the 95% confidence intervals was more than expected by chance.

Several other statistics have been used in the study of extinction clumping within a phylogeny [30], including Moran’s I and the D statistic of Fritz & Purvis [31]. These statistics required the use of a phylogenetic tree, which we do not have currently (but see [21]). It is possible to convert the Linnaean classification into a phylogeny (functions available in the Ape [32] and Geiger [33] packages in R) by collapsing the various taxonomic levels into polytomies and scaling the branches using the stratigraphic durations of terminal taxa [34]. We tried this with our brachiopod taxonomy, but the sheer number of polytomies made the statistics unviable. The D statistic, for example, only functions properly when the resolution on the tree is greater than 70%. Randomly resolving the polytomies [34] and performing the analyses on the randomly resolved trees introduced excessive levels of error and significant results were not obtained.

## Results

**Fig 1:**
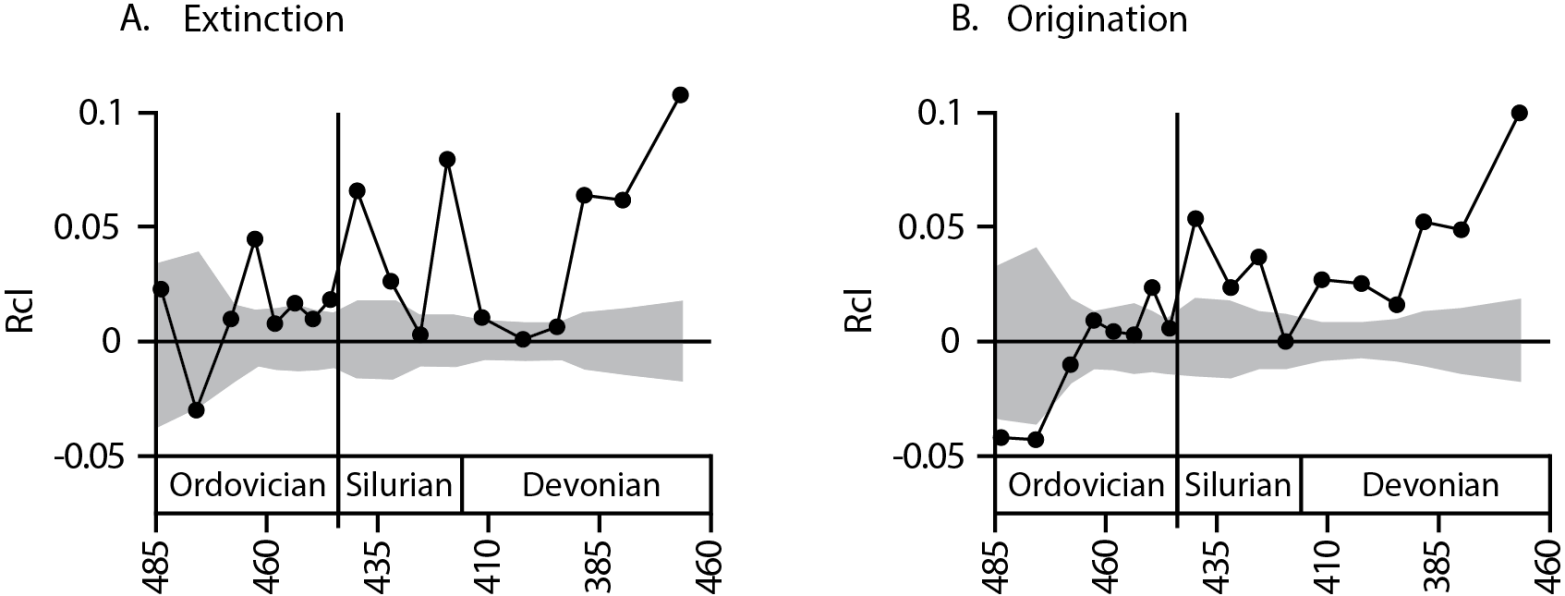
Taxonomic clustering of A. extinction and B. origination for brachiopod genera within families for the Ordovician through the Devonian. Grey region represents 95% confidence intervals around the null distribution of Rcl values determined through randomization.

Analysis of taxonomic clustering for the Middle Ordovician through the Devonian suggests that overall both origination and extinction tend to be clustered within time intervals more than expected by chance (Fig 1). Of eighteen time intervals analyzed for extinction clustering, sixteen time intervals have an Rcl value (the index of taxonomic clustering) greater than zero, suggesting that extinction is usually clustered within families (p = 0.001, exact binomial test). Fourteen of eighteen time intervals are greater than zero for origination, also suggesting that origination is generally clustered within families (p = 0.03, exact binomial test), though a shift from negative to positive values occurs near the end of the Ordovician radiations, potentially suggesting a change in the nature of diversification during this time.

The magnitude of Rcl increases from the Ordovician to the Silurian/Devonian for both extinction (Fig 1A, median_Ordovician_ = .0102, median_Silurian/Devonian_ = .044, KS test *p* = .1) and origination (Fig 1B, median_Ordovician_ = .004, median_Silurian/Devonian_ = .042, KS test *p* = .001). The lack of statistical significance for the increase in extinction clustering likely results from the small number of Ordovician samples (results are significant with the removal of a single large value from the Ordovician) and the conservative nature of the KS test.

**Fig 2:**
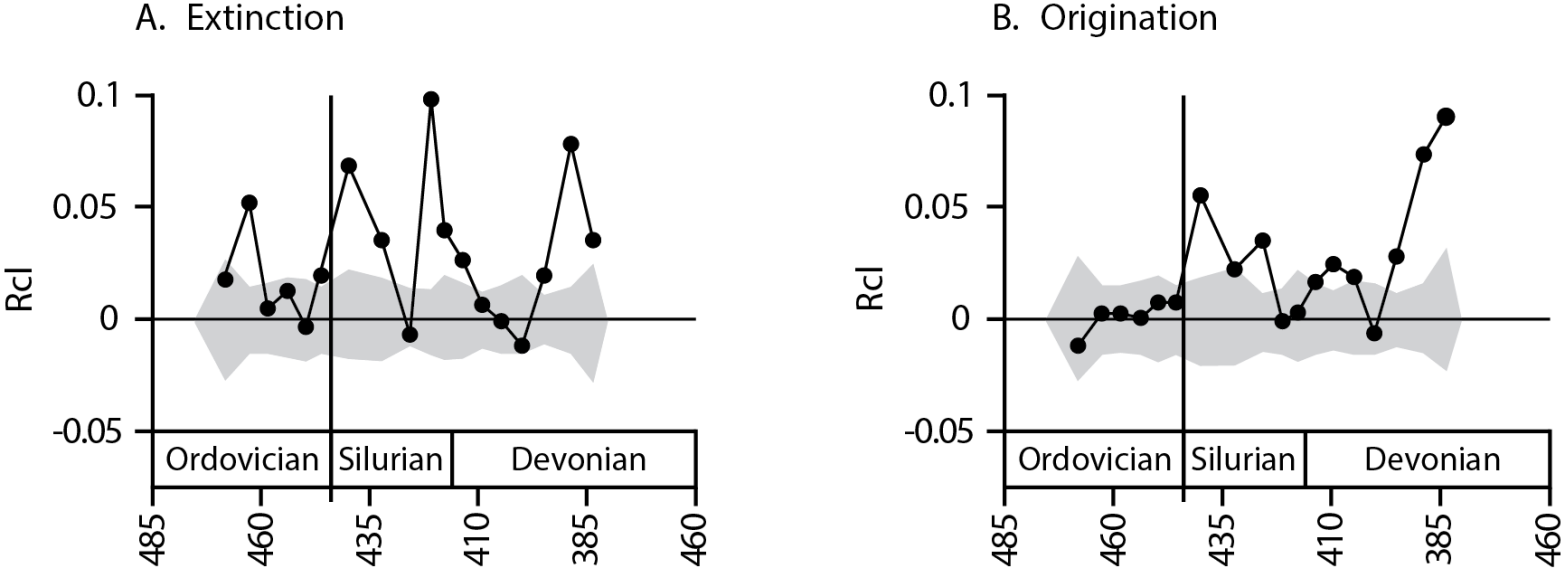
Taxonomic clustering of A. extinction and B. origination for brachiopod genera within substages for the Ordovician through the Devonian. Only intervals with over 100 genera are included in this analysis, as sample sizes smaller than this produce large variations in the null model. Because of this, several early Ordovician and Late Devonian intervals are not included. Results at this finer temporal subdivision largely confirm the results for both families and superfamilies at the stage level, with a shift from predominantly random to predominantly clustered (8 of 12 intervals for extinctions, 9 of 12 for originations) turnover following the Late Ordovician mass extinction.

The increase in Rcl values across the Ordovician-Silurian boundary is accompanied by an increase in the number of intervals that have statistically significant taxonomic clustering of extinction and origination. The increase in extinction Rcl values after the Ordovician is marked by a shift from weakly clustered (or random, with Rcl values near 0) extinctions in the Ordovician to strongly clustered extinctions in the Silurian and Devonian (Fig 1). Of the eight stratigraphic intervals analyzed for the Ordovician, only three have a significant Rcl value for extinctions (i.e. values that stand above the 95% CI for the randomization technique, Fig 1) suggesting that extinctions are only weakly clustered (p = 0.006, exact binomial test). Of these, two stand just slightly outside the null distribution and are similar to values from surrounding stratigraphic intervals. The interval containing the Late Ordovician mass extinction is one of the two moderately significant intervals, suggesting that the mass extinction barely exceeded the expectation of a taxonomically random extinction. The Late Ordovician mass extinction occurred in two pulses separated by 2 myr or less [35], so the temporal resolution of our analyses, which use ~10 myr or ~5 myr bins, is unable to distinguish between these events. However, even if short-lived pulses of extinction varied in the level of clustering, the overall clustering of the mass extinction is what would ultimately govern how much evolutionary history is lost, and is therefore consistent with the lack of ecological severity consistently reported for this event [6,7]. Of the ten stratigraphic intervals analyzed from the Early Silurian through the Late Devonian, however, seven show significantly clumped extinctions, suggesting that extinctions are strongly clustered (p = 8.2 x 10^−8^, exact binomial test) and that a shift in the determinates of extinction risk occurred following the mass extinction. To determine whether bin sizes bias the results, all analyses were repeated for finer bins (generally substages, with a mean duration near 5 Myr), though no such bias is expected *a priori* for these analyses. Subdividing the intervals in this manner resulted in diminished sample sizes for several Late Devonian bins, as the stratigraphic ranges of many genera could not be resolved to substages and were excluded from the analysis. However, for all intervals where sample sizes remained high (N > 100), we found comparable results to those performed at the stage level (Fig 2).

Originations show a pronounced shift from random in the Ordovician to highly clustered in the Silurian and Devonian (Fig 1). Of the eight Ordovician intervals, only one shows significant clustering, and two have significant negative values of Rcl (clustered originations, p = 0.34; dispersed originations, p = 0.06; exact binomial tests). These latter two bins occur close to the end of the Ordovician radiations [36], suggesting the radiations were taxonomically dispersed, though small sample sizes in these time intervals prohibited further analyses. Of the ten stratigraphic intervals analyzed from the Early Silurian through the Late Devonian, nine show significantly clustered originations (Fig 1; p = 1.9 x 10^−11^, exact binomial test), including both of the Silurian intervals representing the post-extinction recovery [37, 38]. In fact, all Lower Silurian intervals show significant clustering of both extinction and origination, suggesting that the shift to clustered origination and extinction resulted from the nature of the recovery rather than the elevated levels of extinction at the end of the Ordovician (see Discussion).

**Fig 3:**
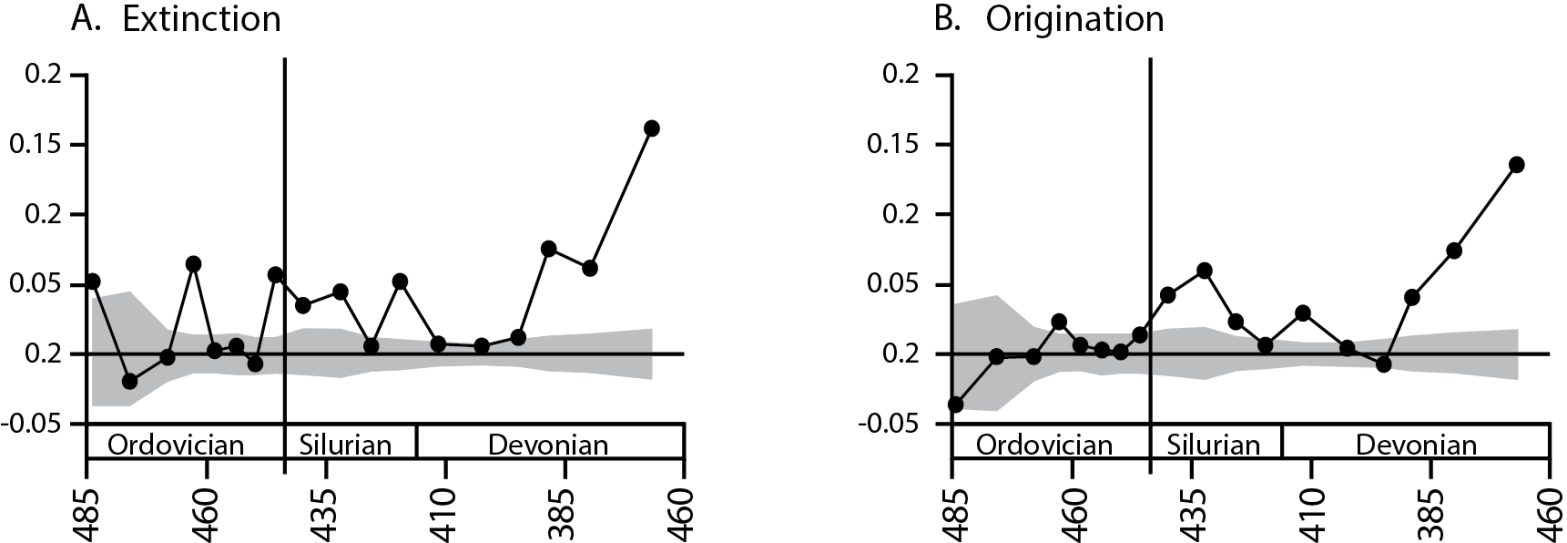
Taxonomic clustering of A) extinctions and B) originations for brachiopod genera within superfamilies for stages ranging from the Ordovician through the Devonian.

Given the extensive taxonomic work on Ordovician-Devonian brachiopods, it is unlikely that the shift in the degree of clustering of origination and extinction within families would reflect purely a taxonomic artifact devoid of any phylogenetic signal. However, because families defined using traditional taxonomic methods may not always represent true monophyletic clades, we repeated the analyses at the next highest taxonomic unit, the superfamily, which is more likely to represent a biological clade. Though the results for individual time intervals vary from the family level, all major results obtained for families hold at the level of superfamily (Fig 3), including the strong shift from weak taxonomically clustered or dispersed origination/extinction before the Late Ordovician mass extinction to strongly clustered origination/extinction after the mass extinction. The consistency of the results at two taxonomic levels suggests the results are robust to potential taxonomic biases.

## Discussion

Origination and extinction of brachiopod genera are taxonomically clustered in most time intervals (Rcl > 0; Fig 1) from the middle Ordovician through the Devonian, a time that spans over 120 million years. Extinction in Mesozoic-Cenozoic bivalves ([12]; origination was not investigated) also was taxonomically clustered throughout a nearly 200 million-year time interval, suggesting that the relationship between extinction prone taxa and phylogeny is a widespread pattern in the history of life.

The Late Ordovician mass extinction was only barely significantly clustered, with an Rcl value similar to those of other Ordovician intervals (Fig 1). These results contrast with those for extinction clustering within marine bivalves for the end-Cretaceous extinction, which was strongly clustered taxonomically as indicated by its Rcl value far above other Cretaceous values [12]. These results support the connection between phylogenetic clustering and the ecological impacts of mass extinctions. An extinction that has a strong phylogenetic signal will eliminate larger proportions of the internal structure of a phylogenetic tree, whereas very little of the tree will be removed from a random extinction regardless of its intensity. In fact, Nee & May [11] estimated that as much as 95% of the modern tree of life will remain even with extinction levels as high as 80%, provided that the extinctions are random with respect to phylogeny. Many traits that promote extinction or origination, including life history or ecological properties, are shared among closely related taxa [12–16]. Therefore, a phylogenetically clustered extinction is more likely to remove ecologically similar taxa, increasing the long-term ecological impact of a mass extinction regardless of the proportion of taxa to go extinct. The weak taxonomic (and, by proxy, phylogenetic) clustering for the Late Ordovician is consistent with the relatively minor ecological consequences documented for this mass extinction [6,7]. The Late Ordovician extinction is among the most severe of the big 5 events, yet produced little long-term ecological or evolutionary effects, with most clades rebounding into similar environments at relatively similar proportions [6]. The lack of phylogenetic clustering would have preserved most of the structure of the global phylogenetic tree and, as a result, most of the ecological or life history traits represented by those clades. The end-Cretaceous was less severe than the Late Ordovician mass extinction when measured as taxonomic severity, but is noted for producing significant ecological impacts [6,7]. The high level of phylogenetic clustering documented for the end-Cretaceous mass extinction [12] could explain this disconnect.

The Late Ordovician mass extinction marks a significant increase in the strength of taxonomic clustering of origination/extinction of brachiopods. Ordovician time intervals showed originations and extinctions that were weakly clustered with respect to taxonomy, with the Ordovician radiations potentially significantly dispersed. Silurian and Devonian time intervals, in contrast, show significant taxonomic clustering within both families and superfamilies. These results again contrast with those of the end-Cretaceous, which showed a shift from taxonomically clustered extinction before the mass extinction to random extinction after the mass extinction [12], presumably caused by the removal of extinction-prone families. For Ordovician-Silurian brachiopods, however, the shift toward clustered extinction and origination occurs in the Early Silurian, following the mass extinction. The long-term shift is therefore likely a result of the nature of the recovery rather than the result of the mass extinction.

The mechanism for such a shift is not immediately clear. The rebound from the Late Ordovician mass extinction was geographically heterogeneous [38], perhaps resulting in clades that were more geographically restricted in the Silurian and Devonian. In fact, the Late Ordovician extinction preferentially eliminated endemic genera living in epicontinental seas [39], as well as genera with ranges constricted to low latitudes [40], perhaps increasing the likelihood that families originating following the extinction would preferentially include genera with restricted geographic ranges. Such a scenario would increase the likelihood that regional perturbations would result in phylogenetically structured extinction [12]. As a preliminary test of this hypothesis, we calculated the average latitudinal ranges of genera within families that occur in the Late Ordovician and Silurian. Paleogeographic data were determined using the Paleobiology Database (paleobiodb.org, data downloaded on 8/12/15). The results for this test show no significant pattern, with the average latitudinal ranges of genera decreasing slightly from the Ordovician into the Early Silurian but then increasing to pre-extinction levels later in the Silurian (S1 Fig). Though the data do not support the hypothesis, such a coarse analysis does not definitively rule out an important role for geographic range in driving the clustered turnover following the mass extinction. More detailed analyses that either A) analyze change in the geographic ranges of genera within families that drove the clustering in the Silurian and Devonian, or B) map geographic range onto a genus-level phylogeny spanning the mass extinction and recovery interval, may prove more effective.

A second potential driver for clumped extinction on a phylogeny is selection for or against phenotypic or life history traits that are inherited by descendant species through time. If selection on these inherited traits was low in the Ordovician but changed as a result of the Late Ordovician mass extinction or Early Silurian recovery, we would expect the change from random to significantly clumped extinction seen in our data. A test for this scenario requires a detailed understanding of the ancestor-descendant relationships of brachiopod genera in order to quantify which traits are inherited and selected for, and therefore the analyses could not be performed at this time.

The explanation for the shift to strong taxonomic clustering of origination in the Silurian and Devonian is less clear, though many of the traits that promote extinction also promote origination, including geographic range, habitat specialization, and body size [41]. Origination rates were substantially higher within pentameride, spiriferide, and athyride brachiopods, all of which increase in diversity in this interval [39]. A concentration of origination in a limited number of groups would cause the Rcl metric to increase.

The causal link between phylogenetic clustering and the ecological consequences of mass extinctions are best evaluated using detailed genus-level phylogenetic relationships among taxa, and must be tested for additional mass extinctions and taxonomic groups. Regardless, the patterns of taxonomic clustering for brachiopods during the Late Ordovician extinction and for bivalves during the end-Cretaceous are both consistent with the hypothesis that phylogenetic clustering of extinction and the subsequent loss of evolutionary history predicts the ecological severity that results from the mass extinction better than simple extinction proportions.

## Conclusions

A strong shift from weak to strong taxonomically clustered origination and extinction occurred following the Late Ordovician mass extinction, yet the mass extinction itself did not show a strongly clustered signal. The shift to strong taxonomic clustering of both extinction and origination occurred in the Early Silurian, suggesting that the nature of the rebound is responsible for the change. These results stand in contrast to results for the end-Cretaceous, which was significantly clustered and, through the elimination of extinction-prone clades, produced a shift from clustered to random extinction patterns. These contrasting patterns of clustered extinction for brachiopods in the Late Ordovician mass extinction and bivalves in the end-Cretaceous mass extinction are both consistent with the hypothesis that the ecological severity of a mass extinction may be related more to the amount of phylogenetic diversity lost than the proportion of taxa lost.

## Supporting Information

S1 Fig The average latitudinal ranges of genera within families containing two or more genera from the Late Ordovician through the Early Silurian. Time intervals correspond to the 10 Myr bins of the Paleobiology Database (paleobiodb.org). ‘Old’ designates families that originated prior to and survived into the interval. ‘New’ designates families that originated within the interval.
(TIF)
